# Natural Drying of Astringent and Non-Astringent Persimmon “Rojo Brillante”. Drying Kinetics and Physico-Chemical Properties

**DOI:** 10.3390/foods10030647

**Published:** 2021-03-18

**Authors:** Cristina M. González, Rebeca Gil, Gemma Moraga, Alejandra Salvador

**Affiliations:** 1 Departamento de Tecnología de Alimentos, Universitat Politècnica de Valencia, Camí de Vera s/n, 46021 Valencia, Spain; crima13c@upvnet.upv.es; 2 Centro de Tecnología Postcosecha, Instituto Valenciano de Investigaciones Agrarias, CRTA, Moncada-Náquera km 4.5, Moncada, 46113 Valencia, Spain; gil_reb@gva.es (R.G.); salvador_ale@gva.es (A.S.)

**Keywords:** *Diospyros kaki*, post-harvest losses, dehydrated persimmon, thin-layer modeling, drying rate

## Abstract

Persimmon (*Diospyros kaki* L.f.) crop has markedly increased in Spain, and “Rojo Brillante” persimmon is the main cultivated variety. This astringent cultivar requires de-astringency treatment before commercialization, which may involve an extra cost. Its short commercial season implies handling large volumes of fruits with consequent postharvest losses. Therefore, the development of derived added-value products is of much interest. In this study, astringent and non-astringent “Rojo Brillante” persimmons were dehydrated by following a natural drying method used in Asia. The drying kinetics and physico-chemical properties were analyzed for 81 days. The results indicated subsequent reductions in weight, water content, and water activity throughout the drying process, and the equatorial diameter decreased. All the employed thin-layer mathematical models were suitable for representing the drying characteristics of both products with similar behavior. The effective water diffusivity values were 5.07 × 10^−11^ m^2^ s^−1^ and 6.07 × 10^−11^ m^2^ s^−1^ for astringent and non-astringent persimmon samples, respectively. The drying treatment significantly decreased the soluble tannins content, and the astringent samples obtained similar values to those obtained for the non-astringent samples in 20 days. The external and internal flesh of the astringent fruit remained orange through the drying period, while brown coloration in the non-astringent fruit was observed after 57 drying days. Therefore, prior de-astringency treatment would not be necessary.

## 1. Introduction

In Spain, persimmon *(Diospyros kaki* L.f.) production has markedly increased over the last 20 years, and the cultivation area has expanded almost 8-fold, from about 2253 ha in 2002 to over 18,000 ha in 2019. As a result, with close to 500,000 tons, Spain is now the second most important persimmon producing country worldwide after China (FAOSTAT, 2018). At present, cultivation is mainly based in “Rojo Brillante” cultivar, with a production of around 429,000 tons in Valencia Community (E Spain) and around 50 tons in Andalusia (S Spain) [1].

“Rojo Brillante” is an astringent persimmon cultivar, which involves its presenting high soluble tannins at harvest [2] and, therefore, the postharvest de-astringency treatment is required before commercialization. The introduction of postharvest techniques based on exposing the fruit to high CO_2_ concentrations to eliminate astringency has been one of the main causes of the expansion of persimmon production in Spain in recent years. With this de-astringency method, it is possible to obtain a fruit without astringency while preserving a firm texture [3,4]. Currently, the Spanish production is mainly destined for exportation markets where there is a demand for persimmon as fresh fruit with a firm texture according to the current quality standards (UNECE 2016) [5]. It is noteworthy that the short commercial season of this cultivar (between mid-October and the end of December) implies the postharvest handling of large fruit volumes with consequent product loss and without achieving the quality required by the fresh fruit market. Therefore, one of the current challenges for the persimmon industry is the search for strategies that increase the value of the discarded fruit.

Drying is a reliable preservation method for fruits in technical feasibility and nutritional quality terms. Unlike expensive energy-intensive artificial drying, natural drying can provide an alternative with adequate drying capacity [6]. Even though natural drying generates a significant loss of bioactive compounds, dried fruit can still be a valuable source of dietary fiber, minerals, and antioxidants. Based on scientific evidence, persimmon can be considered a functional food due to its high contents of bioactive compounds that help reduce the risk of cardiovascular diseases, as well as kidney, colon, and rectal cancer, etc. Hence, dried fruit might be a potential snack that is healthier than most regular snacks [7,8]. In some Asian countries such as China, South Korea, and Japan, dehydrated persimmon is often consumed and commercially produced [9,10,11]. The general procedure followed to make this dried product comprises removing the sepals of the calix and skin, and then hanging the fruit on strings. In China and Japan near the end of the drying period, the dried fruit is kneaded to distribute moisture uniformly in the fruit, and to produce the shape of the final product. However, in South Korea, they are left to hang in a well-ventilated place [12].

Presently, although this drying technology is not applied to “Rojo Brillante”, it would be a good strategy to enhance the surplus fruit and to increase the value of the discarded fruit.

It is necessary to study drying kinetics to know the drying time required to attain a product of adequate quality. Semitheoretical models, based on a serial development of Fick’s second law of diffusion, are the most widely used for food products [13,14,15]. Several studies have focused on drying kinetics in different persimmon formats and varieties, along with different treatments other than drying. García-Pérez, Cárcel, Benedito, and Mulet (2007) [16] and Bozkir et al. (2019) [17] studied the influence of ultrasound or osmo-convective pretreatments on drying cubes of and cylindrical-shaped persimmon. Sampaio et al. (2017) [15] obtained the mathematical model of drying kinetics for the Fuyu persimmon variety in an osmo-convective drying procedure. Çelen et al. (2019) [18] focused on the microwave effects on the drying characteristics of persimmon slices, whereas Doymaz (2012) [19] assessed the drying kinetics and activation energy of persimmon slices using hot air drying. Nevertheless, scarce information on drying kinetics applied in the whole persimmon fruit is reported. Demiray and Tulek (2017) [20] studied the effect of different pretreatments and hot air-drying temperatures on the drying kinetics of whole persimmon. For the specific case of “Rojo Brillante”, no studies have addressed the drying kinetics during the natural drying process of whole persimmon.

In this context, the aim of this research was to study the drying kinetics of persimmon “Rojo Brillante” when the natural drying method (hanging in a well-ventilated place) is applied to astringent and non-astringent fruits (submitted previously to the de-astringency treatment). Moreover, the physico-chemical changes that occur during drying was also studied.

## 2. Materials and Methods

Persimmon fruits “Rojo Brillante” were harvested from commercial orchards in Valencia (E Spain) on 20 December 2018 at a commercial maturity stage (Color index (1000 a/Lb) between 15 and 17; firmness values between 33 and 35 N; initial water content between 77% and 78%). After harvest, the fruits were transported to the Instituto Valenciano de Investigaciones Agrarias (IVIA), where they were carefully selected for uniformity and separated into two groups. The first group was submitted to the astringency removal treatment in closed containers under standard conditions (95–98% CO_2_ for 24 h at 20 °C). The second group was not subjected to the de-astringency treatment. One hundred fruits from both the astringent and non-astringent groups (submitted to the de-astringency treatment) were manually peeled and immersed for 10 min in a 4.5% sodium metabisulfite (Na_2_S_2_O_5_) solution used as a disinfectant. The fruits were individually hung by the pedicel for natural drying in the IVIA pilot plant. In order to study the drying kinetics and the evolution of the physico-chemical properties, one sample of 10 fruits was taken every 17–20 days up to a period of 57 days and at the end of the drying process, two more samples were taken at 67 and 81 days. The sampling dates were as follows: Day 0 (20 December 2018), Day 20 (9 January 2019), Day 40 (29 January 2019), Day 57 (15 February 2019), Day 67 (25 February 2019), Day 81 (11 March 2019).

The average temperature and relative humidity during the drying period were taken from the IVIA weather station, and ranged from 10.3 to 11.3 °C and from 68.6% to 75%, respectively.

### 2.1. Weight Loss and Equatorial, Longitudinal Diameters

The fruits were individually weighed with an Absolute Digimatic caliper (PB3002-S/FACT, Mettler Toledo, Switzerland). The equatorial and longitudinal diameters were measured with a pachymeter (Mitutoyo 500-171-20, Coventry, UK). Ten replicates were performed.

### 2.2. External Color

The external color was evaluated by a Minolta Colorimeter (model CR-300, Ramsey, NY, USA) on 10 fruits. “L”, “a”, “b” Hunter parameters were measured, and the results were expressed as a skin color index: (1000a)/(Lb) [3].

### 2.3. Total Soluble Solids (TSS) and Soluble Tannins (ST)

Three samples of three individual fruits were used to determine TSS and ST. The fruits were cut into four longitudinal parts with the two opposite ends sliced and frozen at -20 °C to determine the ST content. The other opposite fruit parts were placed in an electric juice extractor (model 753, Moulinex, Barcelona, Spain) and filtered through a cheese cloth. The obtained juice was then used to determine the TSS content. ST were evaluated until Day 40 by the Folin–Denis method described by Arnal and Del Río (2004) [21], and the results were expressed as a percent of fresh weight. The TSS juice was measured in triplicate with a digital refractometer (model PR-1, Atago, Japan) and expressed as Brix.

### 2.4. Water Content and Water Activity

Three fruits were individually ground in a crushing machine. The water content and water activity (*a_w_*) were measured using a vacuum oven (Vaciotem-T, J.P Selecta, Abrera, Barcelona, Spain) (60 ± 1 °C and pressure <100 mm Hg) and an Aqualab CX-2 (Decagon Devices Inc., Pullman, WA, USA), respectively. Three replicates were measured per sample.

### 2.5. Mathematical Modeling of Drying Curves

To investigate the drying characteristics of persimmon “Rojo Brillante”, six commonly thin-layer drying semitheoretical models (Table 1) were used to fit the experimental drying data [22,23,24,25,26,27].

The non-linear least squares regression analysis was determined by the statistical software Solver (Excel 2016). In these models, *MR* is the dimensionless moisture ratio in Equation (1):

(1) $$MR=\left(\frac{M_{i}-M_{e}}{M_{0}-M_{e}}\right)$$

where *M_i_* and *M*_0_ are the moisture content (on a dry basis) at any drying time and at the initial time, respectively. *M_e_* is the equilibrium moisture content and is relatively low (about 3%, wb) [28], so it can be neglected. Therefore, *MR* can be expressed as *M_i_*/*M*_0_.

The determination coefficient (*R*^2^) is one of the primary criteria for selecting the best model to define the drying curves. Reduced chi-square (*X*^2^), mean bias error (*MBE*), and root-mean-square error (*RMSE*) are used to determine the quality of fit. These parameters can be calculated using Equations (2)–(4):

(2) $$X^{2}=\;\frac{\sum_{i=1}^{N}{\left(MR_{exp,i\;}-\;MR_{pre,\;i}\right)}^{2}}{N-z}$$

(3) $$MBE=\;\frac{1}{n}\overset{N}{\underset{i=1}{\sum }}\left(MR_{pre,i\;}-\;MR_{exp,\;i}\right)$$

(4) $$RMSE=\;{\left[\frac{1}{n}\overset{N}{\underset{i=1}{\sum }}{\left(MR_{pre,i\;}-\;MR_{exp,\;i}\right)}^{2}\right]}^{1/2}$$

The higher the *R*^2^, and the lower *X*^2^, *MBE,* and *RMSE*, the better the mathematical model fits the experimental data [28]. *MR_exp,I_* is the experimental moisture ratio, *MR_pre,I_* is the predicted moisture ratio, *N* is the number of observations, and *z* is the number of constants.

Another criterion, the relative percent error (PE), is used to evaluate the predictive precision of models [14]. Lower relative PE values give better fitting models (Equation (5)):

(5) $$PE\;\left(\%\right)=\;\frac{100}{N}\overset{N}{\underset{i=1}{\sum }}\frac{\left|MR_{exp,i\;}-\;\left.MR_{pre,i}\right|\right.}{MR_{exp,i}}$$

The drying rate is represented as Δ*M*/Δ*t* (the water content to time ratio to the product’s average water content between two consecutive weight control times) vs. *MR* (dimensionless moisture ratio) [29].

The experimental drying data for determining the effective water diffusivity were interpreted by Fick’s second law of diffusion. To model the total amount of diffusing water entering the astringent and non-astringent persimmon samples, the equation in spheres for “long times” was applied [30]. The effective water diffusivity coefficient (D_e_) was obtained by fitting the corresponding linear equation (Equation (6)), where *r* is the radius obtained from the longitudinal diameter (m), t is the time in days, and *Y* is the reduced driving force defined by Equation (7), in the dry basis moisture content terms.

(6) $$Ln\;Y=\;Ln\left(\frac{6}{\pi^{2}}\right)-\left(\frac{\pi^{2}D_{e}t}{r^{2}}\right)$$

(7) $$Y=\;\frac{M_{i}-\;M_{0}}{M_{e}-\;M_{0}}$$

*M* (g water/g dry solids) at each dehydration (*M_i_*) time in the initial product (*M*_0_) and at the equilibrium time (*M_e_*).

### 2.6. Statistical Analysis

Data were subjected to an analysis of variance (ANOVA) using the least significant difference (LSD) test with a 95% confidence interval to compare the test averages (Statgraphics Centurion XVII Manugistics, Inc., Rockville, MA, USA).

## 3. Results and Discussion

### 3.1. Physico-Chemical Determinations

**Figure 1:**
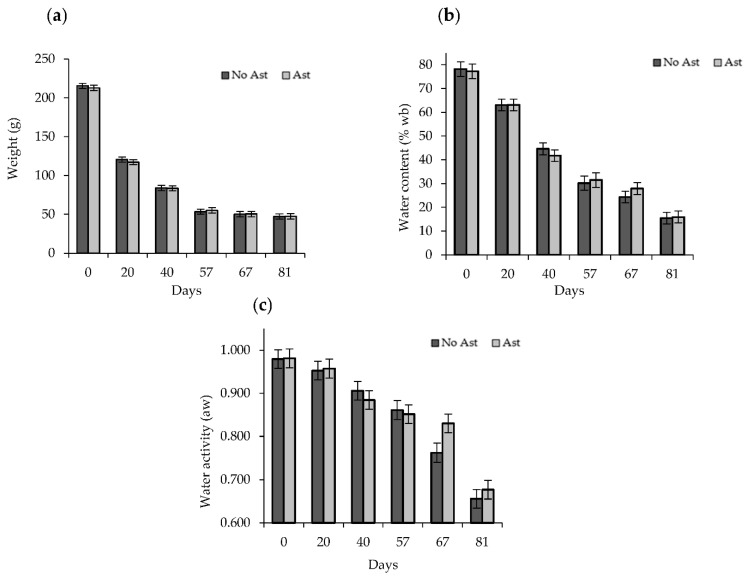
Weight loss (**a**), water content (g/100 g product on a wet basis) (**b**) and water activity (*a_w_*) (**c**) of the non-astringent (no Ast) and astringent (Ast) persimmon samples during the drying treatment. Bars represent the least significant difference (LSD) intervals (*p* ≤ 0.05).

The drying process brought about a marked gradual weight loss for the first 57 days. Thereafter, the fruit weight decreased only slightly until the end of the assay (Figure 1a). Weight loss paralleled the reduced water content (Figure 1b). The water content dropped from 78% at harvest to 25% after 67 days, before lowering to 15% at 81 days. No significant differences in water loss were found between the astringent and non-astringent fruits during the whole study period.

The water activity gradually decreased (Figure 1c) from values of 0.980 on Day 0 to values of 0.860 after 57 drying days, with no differences between the astringent and non-astringent fruits. Unlike the water content, the most marked drop in water activity was detected after 57 days. On Day 67, the *a_w_* of the astringent fruit (0.830) was higher than that of the non-astringent fruit (0.760).

According to previous authors, dried persimmon products are classified as semidried or dried depending on the water content [31]. The final water content of the S Korean semidried and dried persimmons are approximately 50% and 30%, respectively, with drying periods usually lasting 25 days to achieve 50% and approximately 60 days to accomplish 30% [10]. Similarly in our study, on Day 40, fruit samples showed 45% water content, which was 30% on Day 57. After 81 days, a drier product was obtained with 15% water content in both the astringent and non-astringent samples. The drying kinetics of this process could be the key to adjust the drying treatment.

**Figure 2:**
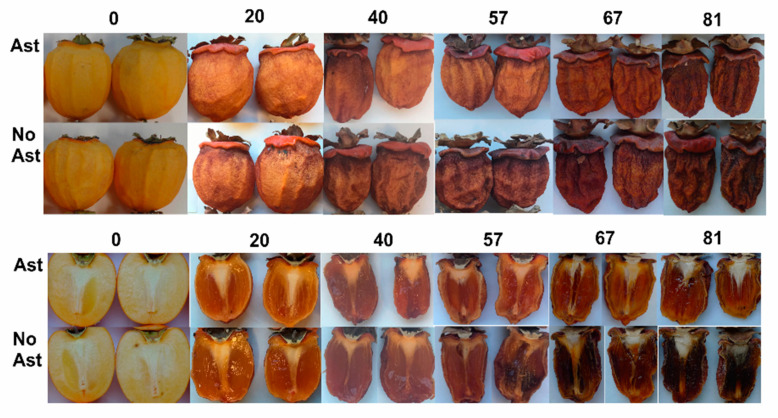
Images of the astringent and non-astringent persimmon cv. “Rojo Brillante” during the drying process.

Figure 2 illustrates the images of the astringent and non-astringent whole persimmon samples, which are cut longitudinally during the drying treatment from Day 0 to 81.

**Figure 3:**
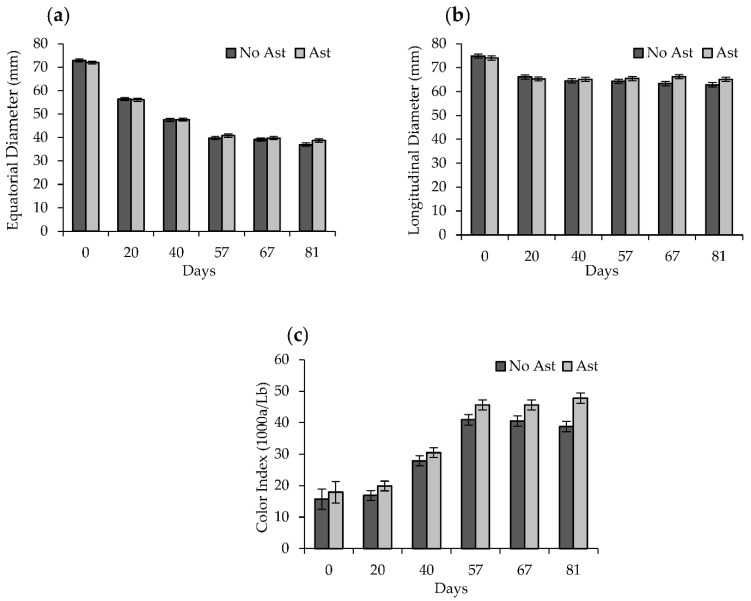
Equatorial diameter (**a**), longitudinal diameter (**b**)**,** and external color index (**c**) of the non-astringent (no Ast) and astringent (Ast) persimmon samples during the drying treatment. Bars represent the least significant difference (LSD) intervals (*p* ≤ 0.05).

The water content loss brought about a major reduction in the equatorial diameter up to 57 days (Figure 3a). The longitudinal diameter slightly lowered after 20 days to remain stable during the subsequent drying periods (Figure 3b). The minor changes in the longitudinal diameter that took place during the drying process were due to the position in which the fruits were hung. No significant differences were observed in the shape changes between the astringent and non-astringent fruits. These changes were accompanied by fruit shrinkage, warping, and wrinkling, which became more evident with the drying time.

During the drying process, the external fruit color darkened (Figure 2). At the beginning of the process, the fruit color index (CI) came close to 18 and reached values near 30 after 40 days with no differences between the astringent and non-astringent fruits. Nevertheless, after 57 days the CI values were significantly higher in the astringent fruit (CI = 46) than in the non-astringent fruit (CI = 40). These differences were still found after 81 days (Figure 3c). The color changes that occurred during the drying period were the result of several biochemical reactions, such as degradation of carotenoids, decomposition of other color pigments, and enzymatic and non-enzymatic reactions [32]. Yamada et al. (2009) [33] suggested that the oxidative and non-oxidative degradation of ascorbic acid would contribute markedly to the browning of this product type, while enzymatic browning, by polyphenol oxidase, and the Maillard reaction, between amino acids and reducing sugars would not play a key role. From Day 67 to 81 of drying, no significant changes (*p* > 0.05) in the color index of the astringent samples were detected, while the non-astringent samples continued to change.

A marked change in the internal flesh structure was also observed after 20 days by showing gelling symptoms, which became much more evident while the drying process prolonged. Mamet, Yao, Li, and Li (2017) [34] reported that persimmon tannins enhance the gel properties of pectin, even though mechanisms remain unclear. It is noteworthy that while the internal flesh color remained orange throughout the drying period in the astringent fruit, the flesh acquired a brown coloration from 57 drying days in the non-astringent fruit. This is consistent with the darker external coloration of non-astringent fruit and the significant difference in water activity at 67 days, which may be related to changes in both structure and water retention capacity.

**Figure 4:**
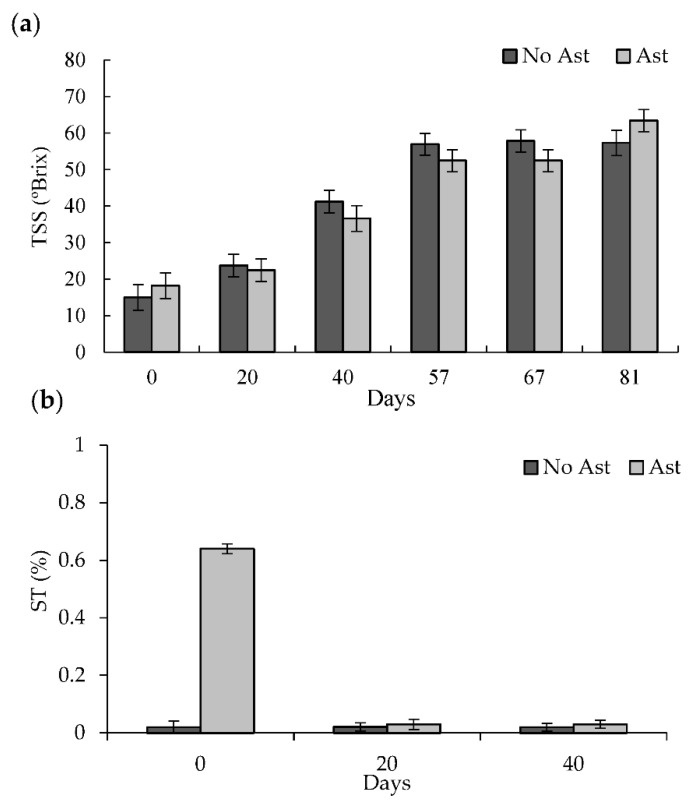
Total soluble solids (TSS) of the non-astringent (no Ast) and astringent (Ast) persimmon samples during the drying treatment (**a**). Total soluble tannin content (ST) of the non-astringent (no Ast) and astringent (Ast) persimmon samples up to the drying treatment at Day 40 (**b**). Bars represent the least significant difference (LSD) intervals (*p* ≤ 0.05).

For TSS (Figure 4a), a gradual increase was observed as the drying process advanced, with values going from close to 18 °Brix on Day 0 to close to 55 °Brix after 57 days, with no significant differences between the astringent and non-astringent fruits. After 81 days, the astringent fruit had higher TSS values (63 °Brix) than the non-astringent fruit (57 °Brix). As drying progressed, soluble solids became concentrated due to the fact that the water loss and new solids were also generated [6]. Similar TSS content have been reported in semidried and dried persimmon from South Korea [10,35].

Initially on Day 0, the astringent persimmons, not previously submitted to the de-astringency treatment, had an ST content of 0.6% (Figure 4b). These values fall within the range found by most previous studies conducted on “Rojo Brillante”, which have been related to high astringency levels in fruits [3,36,37]. In contrast, the non-astringency fruit, submitted to the de-astringency treatment with a high CO_2_ concentration, gave ST content values of 0.02%, which are sensory non astringency values for “Rojo Brillante” [3,36]. After 20 drying days, the ST content values in the astringency fruit notably dropped to 0.03%. The ST values were similar to those of the non-astringent fruit. Tannin insolubilization during the drying process could be associated with structural flesh changes (Figure 2), which happens during natural persimmon fruit ripening [2,32,38]. In astringent cultivars, the ripening process is accompanied by gradual tannin insolubilization, which leads to a progressive decline in ST with subsequent astringency reduction [3]. The softening that occurs during fruit ripening leads to pectin solubilization, which forms a complex with tannins and brings about their insolubilization [37]. Asgar and Yamauchi, Kato (2004) [38] have also related the flesh structural changes found during the sun-drying of Japanese persimmon to the solubilization and depolymerization of pectin polysaccharides.

### 3.2. Fitting of Drying Curves and Drying Rate Determinations

The water content data obtained at the different drying times were converted into a dimensionless moisture ratio (Equation (1)) and then fitted to six thin-layer drying models (Table 1). These models have been used for agricultural products [13,15]. To estimate the parameters from those six models, a non-linear regression analysis was used with both the astringent and non-astringent persimmon samples. The statistical results of the models are summarized in Table 2 (astringent samples) and Table 3 (non-astringent samples). The best models describing the thin-layer drying characteristics of the persimmon samples were chosen with the highest *R*^2^ values and the lowest *X*^2^, *MSE*, *RMSE*, and *PE* values. In both cases, *R*^2^ values were higher than 0.995, while *X*^2^, *MBE*, and *RMSE* were ≤0.001, ≤0.018, and 0.000, respectively. *PE* values were between 2–10% for all the models assessed in both samples. These results were in agreement with those found in fruits such as cape gooseberry [39], pomegranate [40], apple or pumpkin [13].

**Figure 5:**
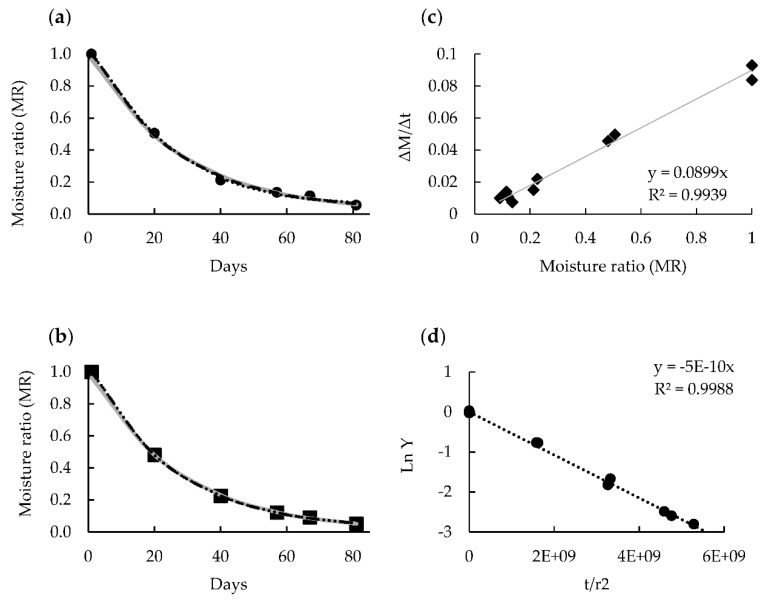
Modeling the drying curves of the astringent (**a**) and non-astringent (**b**) persimmon fruits with the Midilli (dotted line), Logarithmic (dashed line), and Verma models (gray line). Drying rate of the astringent and non-astringent persimmon samples (**c**). Effective water diffusivity (*D_e_*) determination by equation $Ln\;Y=\;Ln\left(\frac{6}{\pi^{2}}\right)-\left(\frac{\pi^{2}D_{e}t}{r^{2}}\right)$ in the astringent and non-astringent persimmon samples (**d**).

Figure 5a,b shows the Midilli, Vermal, and Logarithmic models selected to represent the drying characteristics of the whole astringent and non-astringent persimmon samples. After fitting the experimental data, it took 34 days to reach a water content of 50% and 57 days to obtain 30%. Under these conditions, the desirable semidried and dried persimmon would be obtained. Figure 5c shows the effect of Δ*M*/Δ*t* vs. *MR* on the drying rate of the astringent and non-astringent samples, where a good correlation was obtained (*R*^2^ = 0.9855). In both the sample types, the drying rate was rapid during the initial period, but then slowed down in the later stages, and no constant rate of the drying period was observed. The entire drying process occurred during the falling-rate period. When the decrease in the drying rate was linear with the water content, water evaporation in the material continued as during the constant rate period. This indicates that mass transfer is governed by intrinsic product properties and internal resistance to water diffusion to the surface [41]. This result was similar to those reported for the thin-layer drying of other biomaterials [29,42,43].

In Figure 5d, *Ln Y* vs. *t*/*r*^2^ is plotted to determine, from the slope (*π*^2^*D_e_*), the effective water diffusivity of both the astringent and non-astringent persimmon samples. Fick’s second diffusion law has been widely used to describe the drying process during the falling-rate period for biological material [44]. The effective water diffusivity results were similar in both the astringent and non-astringent persimmon samples, with values of 5.07 × 10^−11^ and 6.08 × 10^−11^ m^2^ s^−1^, respectively. The *R*^2^ was 0.996. The *D_e_* values fell within the general range of 10^−12^–10^−8^ m^2^ s^−1^ in food materials [45]. Similar results were reported by Doymaz I (2012) [19] in persimmon slices (between 7.05 × 10^−11^ and 2.34 × 10^−10^ m^2^ s^−1^).

## 4. Conclusions

For the first time, the kinetics during the natural drying method followed in Asian countries (hanging the whole fruit in a well-ventilated place) was studied in “Rojo Brillante” persimmon. Since “Rojo Brillante” is an astringent cultivar, the behavior of the astringent and non-astringent fruits (submitted to the CO_2_ treatment) was compared. The used thin-layer mathematical models were suitable for fitting the drying kinetics. No significant differences between the astringent and non-astringent fruits were found. Around 34 days were needed to reach a final water content of 50% and 57 days to reach one of 30%. This drying treatment was able to produce a natural decrease in ST contents. The astringent and non-astringent fruits obtained similar values in just 20 days. Different behaviors between the astringent and non-astringent samples were observed in *a_w_*, external and internal color at 57 drying days. The astringent fruit remained orange, while brown coloration developed on the non-astringent fruit. Hence, the de-astringency treatment is not recommended. This natural drying technology, not yet applied to the “Rojo Brillante” persimmon industry, could be a good strategy to enhance the surplus of this seasonal fruit. 

## Figures and Tables

**Table 1:** Mathematical models given by several authors for drying curves.

Model	Mathematical Equation	References
Newton	$MR=\exp\left(-kt\right)$	[22]
Page	$MR=\exp\left(-kt^{n\;}\right)$	[23]
Midilli et al.	$MR=a\exp\left(-kt^{n}\right)+bt$	[24]
Logarithmic	$MR=a\;exp\;\left(-kt\right)+c$	[25]
Henderson and Pabis	$MR=a\exp\left(-kt\right)$	[26]
Verma model	$MR=a\;exp\;\left(-kt\right)+\left(1-a\right)\exp\left(-gt\right)$	[27]

*k*, *n*, *a*, *g*, *c*, *b*: Constants of each model applied; *t*: Time in days.

**Table 2:** Values of the parameters of the models for astringent persimmon “Rojo Brillante”.

	Models Parameters						Statistical Parameters				
Models	*k*	*n*	*a*	*g*	*c*	*b*	*R* ^2^	*X* ^2^	*MBE*	*RMSE*	*PE*%
Newton	0.035	-	-	-	-	-	0.997	0.001	0.017	0.000	7.071
Page	0.030	1.043	-	-	-	-	0.997	0.001	0.017	0.000	9.021
Midilli et al.	0.027	1.101	1.028	-	-	0.000	0.998	0.000	0.011	0.000	10.243
Logarithmic	0.039	-	1.022	-	0.019	-	0.998	0.000	0.012	0.000	7.965
Henderson and Pabis	0.037	-	1.037	-	-	-	0.998	0.000	0.012	0.000	7.586
Verma model	0.036	-	0.007	0.036	-	-	0.998	0.001	0.018	0.000	7.663

*k*, *n*, *a*, *g*, *c*, *b*: Constants of each model applied. *R*^2^: determination coefficient; *X*^2^: reduced chi-square; *MBE*: mean bias error; *RMSE*: root-mean-square error; *PE*%: relative percent error.

**Table 3:** Values of the parameters of the models for non-astringent persimmon “Rojo Brillante”.

	Models Parameters						Statistical Parameters				
Models	*k*	*n*	*a*	*g*	*c*	*b*	*R* ^2^	*X* ^2^	*MBE*	*RMSE*	*PE* *%*
Newton	0.037	-	-	-	-	-	0.999	0.000	0.008	0.000	2.305
Page	0.032	1.040	-	-	-	-	0.999	0.000	0.010	0.000	4.632
Midilli et al.	0.041	0.983	1.042	-	-	0.000	0.999	0.000	0.002	0.000	1.943
Logarithmic	0.039	-	1.028	-	0.011	-	0.999	0.000	0.002	0.000	2.032
Henderson & Pabis	0.038	-	1.037	-	-	-	0.999	0.000	0.004	0.000	3.095
Verma model	0.037	-	0.017	0.037	-	-	0.999	0.000	0.008	0.000	2.305

*k*, *n*, *a*, *g*, *c*, *b*: Constants of each model applied. *R*^2^: determination coefficient; *X*^2^: reduced chi-square; *MBE*: mean bias error; *RMSE*: root-mean-square error; *PE*%: relative percent error.

## Data Availability

Not applicable.
